# National and subnational analysis of self-reported diabetes mellitus and health inequalities in Europe

**DOI:** 10.1038/s41598-026-49349-7

**Published:** 2026-05-29

**Authors:** Carlos Alexandre Soares Andrade, Nour Mahrouseh, Nóra Kovács, Sarah Cuschieri, José Chen-Xu, Orsolya Varga

**Affiliations:** 1 Department of Public Health and Epidemiology, Faculty of Medicine, University of Debrecen, Debrecen, Hungary; 2 Department of Periodontology, Oro-Dental and Implant Surgery, Faculty of Medicine, University of Liège, Liège, Belgium; 3 Faculty of Medicine and Surgery, University of Malta, Msida, Malta; 4 Department of Epidemiology and Biostatistics, Western University, London, Canada; 5 NOVA National School of Public Health, Public Health Research Centre, Comprehensive Health Research Center, CHRC, REAL, CCAL, NOVA University Lisbon, Lisbon, Portugal

**Keywords:** Health inequality, Europe, Diabetes mellitus, Prevalence, Diabetes, Health care

## Abstract

Diabetes mellitus (DM) has been a major public health concern with increased epidemiologic and economic burden in Europe. We aimed to estimate self-reported national and subnational DM prevalence and inequalities in individuals aged 50 years and over in 13 European countries between 2010 and 2019. Microdata were extracted from waves 4 (2010/2011), 5 (2013), 6 (2015), 7 (2017/2018) and 8 (2019) of the Survey of Health, Ageing and Retirement in Europe (SHARE) database. The analysis was conducted at both national and subnational levels, comprising 13 European countries. Health inequalities were assessed using the gini coefficient (GC) and concentration index (CI). Socioeconomic risk factors were included in the inequality analysis (urbanization, income, and educational level) in addition to multilevel regression model. The prevalence of DM decreased from 11% in wave 4 to 10.6% in wave 8, with the lowest recorded in Switzerland at 5.9% in wave 5 and the highest observed in Czechia at 16.7% in wave 8. DM prevalence varied widely subnationally, ranging from 2.1% in wave 8 Zentralschweiz (Switzerland) to 28.5% in wave 8 in Střední Čechy in Czechia. The GC demonstrated an increase from 21.9% (95% CI 17.0, 24.5) in wave 4 to 22.5% (95% CI 19.4,25.2) in wave 8, with Switzerland exhibiting the highest coefficient in wave 8 [30.1% (95% CI 7.9, 41.7)]. The CIs for income and educational level at national level were consistently low and mostly negative. The education and income CI ranged from [− 0.0182 (95% CI – 0.0242, − 0.0122) to − 0.0010 (95% CI − 0.0021, − 0.0001)] and [− 0.0278 (95% CI − 0.0411, −0.0166) to − 0.0018 (95% CI – 0.0032, – 0.0005)] across waves 4 and 8, respectively. The CI for urbanization displayed both negative and positive values, but most were not statistically significant (*p* > 0.05). Inequalities exist in the self-reported prevalence of DM both nationally and subnationally, with elevated rates observed among individuals with lower educational and income levels. Addressing these significant health inequalities calls for the development of prevention programs and policies by the European countries and subnational regions, specifically tailored to prioritize and support vulnerable groups.

**Supplementary Information:**

The online version contains supplementary material available at 10.1038/s41598-026-49349-7.

## Introduction

Diabetes Mellitus (DM) is currently acknowledged as a major public health concern worldwide. The increase in DM burden is due to a wide network of lifestyle, socioeconomic and genetic determinants. Associations between prevalence of DM and socioeconomic risk factors such as income, education level and urbanization have been extensively investigated^1^. Individuals with lower incomes, lower educational levels, and those living in urban areas face higher diabetes risks due to barriers in accessing quality healthcare, higher rates of food insecurity, and unhealthier lifestyle^2,3^. For this reason, understanding the prevalence of DM and its association with socioeconomic and environmental risk factors is important for identifying inequalities in the burden of disease.

Primary prevention, which involves addressing lifestyle risk factors, is regularly featured on the policy agenda in European Union (EU) countries as DM is an expensive condition to manage. Beyond the treatment cost of the disease, the micro- and macrovascular complications of this chronic disease represent immense indirect costs and decreasing quality of life. Individuals with DM require greater attention in terms of healthcare spending, as they utilize health services more frequently and require more medication than non-diabetic individuals. The economic sector experiences indirect consequences as individuals with DM are more prone to taking sick leaves, leading to a reduction in overall work productivity^4,5^. Furthermore, DM was accountable for more than 4 million deaths worldwide among adults aged 20–79 years, which represents 11.3% of deaths from all causes in 2019^6^. The number of deaths attributable to DM for this age group was 465,916 only in Europe^6^. DM is proven to be one of the 10 most common causes of death in adults^7^, although the type 2 DM mortality trends are decreasing in the past 30 years in EU^8^.

The prevalence rates of DM risk factors both within and between nations are strikingly unequal. Many studies report high level of inequalities when DM is assessed across regions of the former German Democratic Republic in comparison to south Germany, or between eastern and south-western Finland, or in regions of England^9–12^. When comparing incidence and prevalence within regions of EU, studies reported that they are higher in South and East than in West. This can be explained by different healthcare systems, socioeconomic status, and lifestyle^13^. Educational level, socioeconomic factors, health system, lifestyle, smoking behavior, and genetics stand out as diverse variables at national and regional level^9^.

Studies comparing countries are typically constrained to the national average, which may obscure disparities. Subnational analysis in the EU is based on the Nomenclature of territorial units for statistics (NUTS) classification. It is a system that has divided the EU according to hierarchical and economic instruments - and the original purpose of this division was to collect and develop regional statistics, to carry out socio-economic analyses at regional level and to analyze EU regional policies^14,15^. The latest NUTS classification was created in January 2021 and comprises 92 regions at NUTS 1, 242 regions at NUTS 2 and 1166 regions at NUTS 3 level^16^. Health indicators vary significantly within a country, and utilizing the NUTS classification enables addressing them at regional levels. Socioeconomic, educational, lifestyle, and dietary factors may account for regional variations in DM burden within NUTS-level regions. Based on a study analysing 2019 data, DM and its comorbidity present a different burden across NUTS regions in EU countries. EU regional policies should be considered in addressing these differences^17–20^.

National averages and single-wave studies can obscure where diabetes burden is concentrated within countries and how inequalities change over time^21^. A multi-wave NUTS-level analysis across a fixed set of European countries is therefore needed to quantify geographic and socioeconomic inequalities, provide uncertainty estimates, and assess robustness to underdiagnosis. The aim of the present study is to estimate self-reported prevalence of DM across countries and NUTS regions in 13 EU countries between 2010 and 2019. In addition, the study estimates socioeconomic inequality using Gini Coefficient (GC) and Concentration Index (CI) in DM prevalence and examines association between DM with risk factors across EU countries and NUTS regions.

## Materials and methods

### Data source

The present study collected self-reported data from the Survey of Health, Ageing and Retirement in Europe (SHARE) database. SHARE is a 9-wave self-reported social science panel study of microdata from individuals aged 50 and over in European countries. The survey was launched in 2004, and it was conducted approximately every 2 years: wave 1 in 2004/5, wave 2 in 2006/7, wave 3 in 2008/9, wave 4 in 2010/11, wave 5 in 2013, wave 6 in 2015, wave 7 in 2017, wave 8 in 2019/2020, and wave 9 in 2021/2022. In summary, SHARE data comprises 18 years of longitudinal microdata, for more than 160,000 people in 28 European countries and Israel, regarding effects of health, socioeconomic, educational, and environmental policies over the life-course of European individuals^22^.

The survey included not only the national demographic region of each participant, but also subnational level information on NUTS1, NUTS2, and NUTS3 levels for most participants, where applicable. The data collection was performed by trained interviewers using standardized computer-assisted methods in which self-reported answers were given face-to-face. This project was approved in the Ethics Council of the Max Planck Society and Ethics Committee of the University of Mannheim; also, a consent form was signed by all interviewees. Furthermore, the SHARE database was originally funded by the European Commission as a result of the 5th, 6th and 7th Framework Programs for Research. Other funding institutions also joined the initiative to support SHARE data collection in later stages, such as U.S. National Institute on Aging and the German Ministry of Education and Research.

### Population

The present study extracted repeated cross-sectional self-reported microdata from waves 4 (*n* = 49655), 5 (*n* = 60507), 6 (*n* = 57082), 7 (*n* = 49588) and 8 (*n* = 29621) of the SHARE database (Supplementary file 1). Those waves were chosen as DM related questions differed for earlier waves affecting longitudinal comparability. To ensure consistent measurement and robustness of prevalence and inequality estimates, only waves with harmonized and comparable DM variables were included in the analysis. Thirteen European countries that were available in all chosen waves were included to facilitate consistent assessment over time. The countries present in those waves were: Austria, Belgium, Czechia, Denmark, Estonia, France, Germany, Italy, Netherlands, Slovenia, Spain, Sweden, and Switzerland. In addition, DM prevalence was examined at the NUTS regional level. The following individuals were excluded: (1) under 50 years old, (2) non-residents of one of the 13 included countries, (3) those with missing DM data and corresponding sampling weight. We defined the DM onset if the individual responded “yes” to at least one of the two questions: (1) “Has a doctor ever told you that you had diabetes or high blood sugar / Do you currently have diabetes or high blood sugar?” or (2) “Do you currently take drugs at least once a week for diabetes or high blood sugar?”. This means that all the individuals who responded “no” to both questions, were considered non-diabetic. Self-reported DM may include both type 1 and type 2 DM; however, given the substantially higher prevalence of type 2 DM in this age group, most reported cases are likely attributable to type 2 DM. For the purposes of the analysis, all cases were therefore treated as type 2 DM. Details of frequency distribution of participating individuals are available in Supplementary file 1. Prevalence of DM was calculated by dividing the number of DM individuals at each wave by the number of interviewed individuals using sampling weights, except for prevalence of the Netherlands in waves 6 and 7, sampling weights were unavailable, each individual was assigned a weight of one. DM prevalence for each country was also calculated for 5 age groups (50–59, 60–69, 70–79, 80–89 and, 90 and above) Prevalence data was not disaggregated by sex.

### Analysis of health inequalities

Many statistical analyses have been used across research history to measure inequalities in health. The GC and CI are part of the Lorenz curve family, and they are widely accepted in a variety of fields including economics, education, policy, and health^23^.

The Lorenz curve is a graphical representation in curve shape that represents the cumulative frequency of a variable in comparison to a uniform distribution (equality). When applied to the health field, the Lorenz curve is usually comprised by two axes, (x) cumulative proportion of the population ranked by health and (y) cumulative proportion of health variable in the population. Also, a hypothetical 45° diagonal line is drawn, representing a perfect distribution of health within the population. The Lorenz curve is a deviation plotted between the hypothetical line and the axes x and y, and this curve represents the level of inequality; since the greater the deviation of the curve in relation to the line, the greater is the inequality. The line also represents neutrality regarding the chosen health variable, considering that curves above the line represent prejudicial variables to the population and those below the line represent beneficial variables^23,24^.

GC is a measure to analyze pure inequality, and it can apply comparisons between countries or subnational regions. In summary, the GC is a measure for the extent of inequality, so it is calculated based on the area between the 45° line and the Lorenz curve. This coefficient ranges from 0 to 1. As 0 represents perfect equality, i.e., a hypothetical society in which every individual of a population receives exactly the same healthcare or has the exact same level of health. In this scenario, 1 represents total inequality, a society in which only one individual receives all the available healthcare or is the only one to retain all health. In the last scenario, the healthcare services or level of health is assumed to be equally distributed for the whole included population^23,25^. The health measure chosen in this study to calculate the GC was the prevalence of DM in individuals of a population.

Differently from the GC, the CI measures socioeconomic factors related to health. This index is measured in a similar method as the GC, but it ranges from − 1 to + 1, in which the negative values represent curves above the diagonal line and positive values are below it^23,25^. Three variables were included in the statistical analysis in order to address all the possible explanations for the differences in DM prevalence and health inequalities. The included geographical nominal variables were inserted in the model as country, NUTS 1, NUTS 2, or NUTS 3. Total income was included as a continuous variable, ranked from lowest to highest, urbanization was included as binary variable (rural (0) and urban (1)), and educational level was included in the analysis as an ordinal variable, ranked as from lowest to highest (none/primary (lowest), secondary, and tertiary (highest)).

### Statistics

We calculated the prevalence for each country based on NUTS 2 where applicable, and NUTS 1 and NUTS 3 based on data availability with minimum limit of 50 observations in a region, using sampling weights. Sampling weights were used via svydesign() function from the survey package incorporating individual-level sampling weights and no clustering variable was specified. For wave 6 and 7, sampling weight for the Netherlands was considered as 1 in all relevant calculations. Missing data were imputed for education, urbanization, and income using multiple imputation by chained equations (MICE)^26^. Missing data of DM and sampling weights were not imputed and were not included in the sample; the final sample size was 246,453. Our research objective is to evaluate the inequality in DM prevalence among 13 European countries. We calculated the GC for each country, using prevalence of relevant NUTS. However, we incorporated NUTS 1 (Germany and Netherlands), NUTS 2 (Austria, Belgium, Czechia, Denmark, France, Italy, Spain, Sweden and Switzerland), and NUTS 3 (Estonia and Slovenia) in our analysis depending on data accessibility and the number of NUTS per region. We utilized the R package “ineq” with bootstrapping, we were able to obtain more robust and reliable estimates of the GC for each country and their corresponding confidence intervals.

We calculated CI using individual data based on Erreygers concentration index^27^ and investigated the association between DM presence and socioeconomic factors, including income, education, and degree of urbanization. The CI is a widely used measure of health inequality that allows us to assess the degree to which a particular health outcome is concentrated. In this case, DM cases were concentrated among different socioeconomic groups. Education was categorized into three levels ranked from lowest to highest (primary education (lowest), secondary education and tertiary education (highest)). Household income per month was used as a continuous variable, ranked from lowest income to highest. The degree of urbanization was divided into two categories: rural (0) and urban (1). Sampling weight were used, except for CI for European (13), CI for Netherlands as country and NUTS1 for wave 6 and 7 as the weight for Netherlands was considered as one. Bootstrapping was carried out to obtain more robust and reliable estimates of CI.

Moreover, descriptive statistics were carried out to assess DM, sex, age groups, income, level of education, and urbanization. Multilevel logistic regression model was conducted to assess the relation between income, urbanization and education while controlling for sex and age. Country was considered as random effect, and model outputs were presented in odds ratios (OR).

Sensitivity analysis was carried out to account for undiagnosed DM in self-reported surveys, by applying a correction factor sourced from the International Diabetes Federation (IDF) Atlas 11th edition that states that 33.6% of adult diabetes cases are undiagnosed in Europe^28^. We calculated an adjusted prevalence by dividing self-reported prevalence by 0.664 under the assumption of self-reported DM in the SHARE survey captures 66.4% of true cases.

P-values smaller than 0.05 were considered statistically significant. All analyses and maps were performed and produced using R 4.5.2. Prevalence estimates were calculated using weighted survey analysis with the “survey” package in R. Gini coefficient was assessed using the “ineq” package and Erreygers concentration index was implemented with bootstrap confidence intervals using (boot package) to quantify socioeconomic gradients.

## Results

**Fig. 1:**
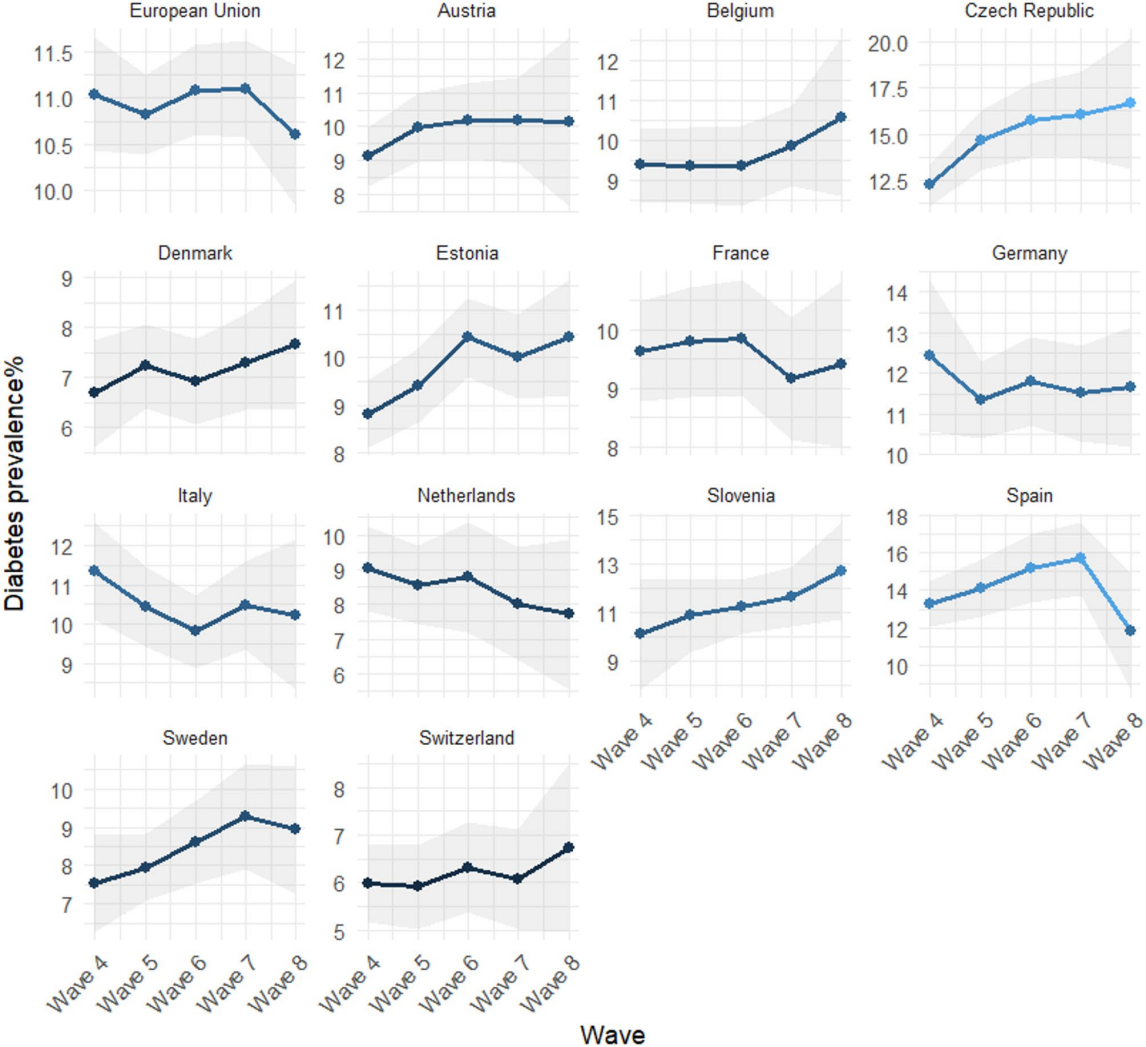
DM prevalence (%) for waves 4, 5, 6, 7, and 8 in Europe (13) and each individual country. Grey area represents 95% confidence interval (95% CI).

The prevalence of DM across the 13 countries included in this study was 11% (95% CI 10.4, 11.6) in wave 4, 10.8% (95% CI 10.4, 11.3) in wave 5, 11.1% (95% CI 10.6, 11.6) in wave 6, 11.1% (95% CI 10.6, 11.6) in wave 7, and 10.6% (95% CI 9.8, 11.4) in wave 8 (Fig. 1 and Supplementary file 2). The highest prevalence was observed in Czechia, where it increased from 12.3% (95% CI 11.1, 13.3) to 16.7% (95% CI 13.1, 20.2). The lowest prevalence percentages were found for Switzerland and Denmark. Switzerland started as the lowest prevalence in wave 5 with 5.9% (95% CI 5.0, 6.8), but it progressively increased to 6.7% (95% CI 5.0, 8.5) in wave 8. Denmark presented its lowest percentage in wave 4 with 6.7% (95% CI 5.6, 7.8) of DM prevalence, increasing to 7.7% (95% CI 6.4, 9.0) in wave 8. Adjusted DM prevalence based on IDF correction factor is presented in Supplementary file 2. Country specific prevalence divided by 5 age groups (50–59, 60–69, 70–79, 80–89, 90 and above) is presented in Supplementary file 3.

### DM prevalence at the level of NUTS regions

**Fig. 2:**
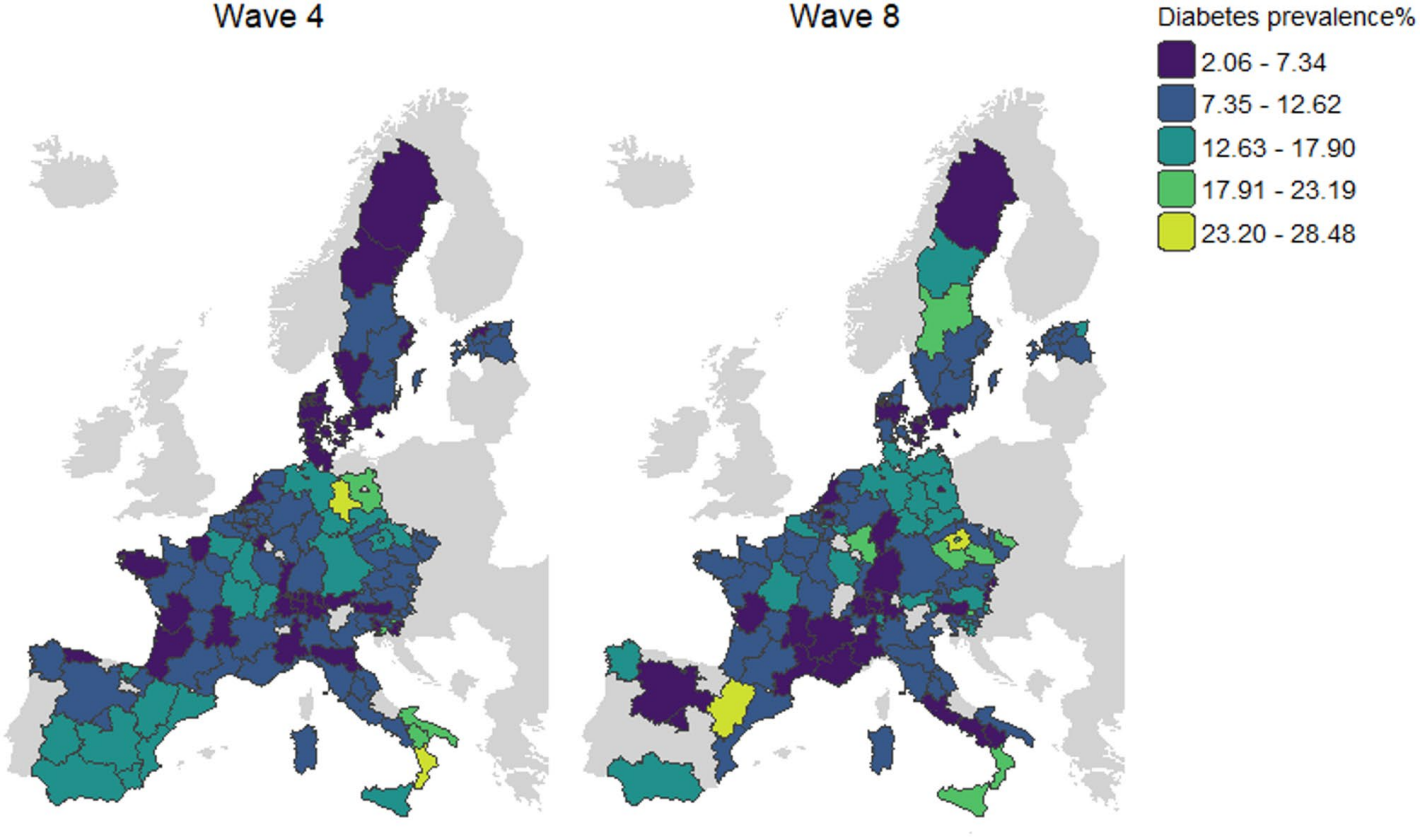
DM prevalence percentages across the NUTS regions of 13 European countries for waves 4 as the base and 8.

The DM prevalence for NUTS regions ranged from 2.1% (95% CI 0.3, 3.8) Zentralschweiz (Switzerland) in wave 8 to 28.5% (95% CI 0.3, 56.7) in wave 8 in Střední Čechy in Czechia. Other regions with the highest prevalence percentages over the study period included: Calabria in Italy and Sachsen-Anhalt in Germany, 26.5% (95% CI 18, 34) and 25.5% (95% CI 11.9, 39.0), respectively. The lowest prevalence was observed in Piemonte, Italy: 2.4% (95% CI 1.0, 3.8). Zentralschweiz, Switzerland, also had among the lowest prevalence estimates across waves: 2.6% (95% CI: 0.9, 4.2) in wave 4, increasing to 3.1% (95% CI 1.1, 5.1) in wave 6, and then declining to its lowest value in wave 8. Maps for waves 4 and 8 are shown in Fig. 2, and detailed results for waves 4–8 are provided in Supplementary File 4.

### Overall health inequality - Gini coefficient

**Fig. 3:**
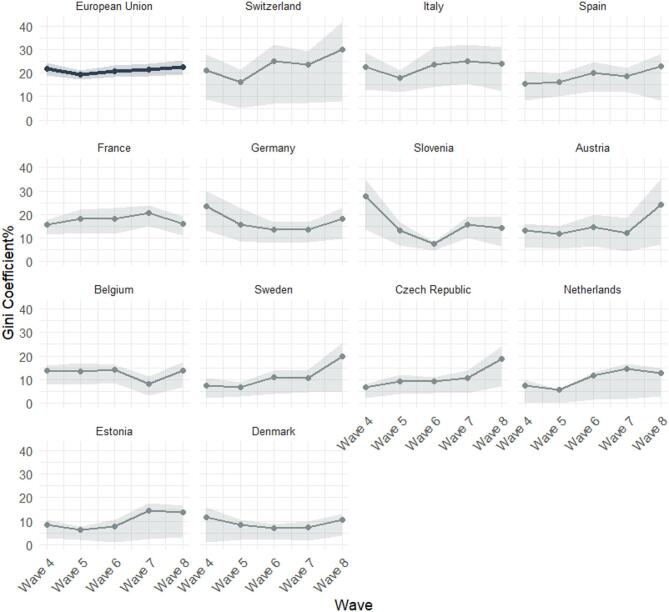
Gini coefficient for 13 European countries included in the analysis in waves 4, 5, 6, 7 and 8. The Gini Coefficients are ordered by the average Gini of each country in a descending order to show highest inequality first. Grey area represents 95% Confidence Interval (95%CI).

The overall European (13) GC was 21.9% (95% CI 17.0, 24.5) in wave 4, 19.2 (95% CI 17.1, 21.2) in wave 5, increasing progressively to 20.8% (95% CI 18.1, 23.1) in wave 6, to reach 22.5% (95% CI 19.4, 25.2) in wave 8, (Table 1). The highest GC was found in Switzerland in wave 8, 30.1% (95% CI 7.9, 41.7). A few other high values were found in Slovenia in wave 4 and Italy in wave 7, 27.6% (95% CI 13.70, 34.74) and 24.9% (95% CI 15.31, 32.07), respectively. The lowest GC was found in the Netherlands and Estonia, with Estonia presenting 6.33% (95% CI 2.01, 7.67) in wave 5 and the Netherlands 5.8% (95% CI 0.08, 5.8) in wave 5. Czechia, Denmark, Estonia, and Sweden presented the lowest GC values (Fig. 3 and Supplementary file 5).

### Health inequality among socioeconomic groups - Concentration index

**Fig. 4:**
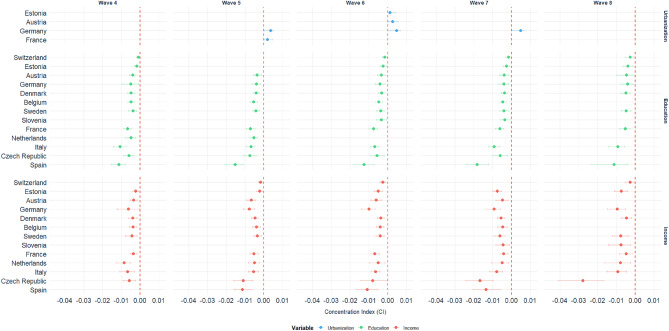
Concentration Index for urbanization, income, and educational level for significant countries included in the analysis for waves 4, 5, 6, 7, and 8. Points represent country-specific Erreygers concentration indices (CI) for diabetes for statistically significant countries by urbanization, education, and income across waves (4,5,6,7, and 8) with horizontal bars indicating 95% confidence intervals (95% CI). The vertical dashed line at zero represents zero inequality.

The CI presented the highest values for urbanization in relation to educational level and income (Table 1). The pooled data of EU 13 showed small statistical significance of inequalities in educational level and income across all waves as it indicates that DM is concentrated among individuals with lower levels of education and lower income. In terms of urbanization, DM was concentrated among urban areas compared to rural areas in all waves except for wave 4 (not significant). Country specific analysis presented in terms of educational level inequalities that concentration was relatively small and negative, indicating that DM is concentrated in low levels of education. The highest CI value, indicating high but still relatively small inequality, was observed in Spain wave 7 (CI:−0.0182; 95% CI −0.0242, − 0.0122) and lowest, presenting low inequality, was present in Switzerland for all waves; lowest in wave 6 (CI:−0.0014; 95% CI −0.0028, − 0.0003). Only one positive CI was found in Slovenia wave 5 (CI: 0.0015, 95% CI -0.0023, 0.0061) but it was not statistically significant (Fig. 4 and Supplementary file 6). When comparing NUTS regions, the CI for education level the highest concentration was found in Champagne-Ardenne region in France (CI:-0.0516, 95% CI -0.1149, -0.0126), indicating concentration of DM in low education levels followed by Aragón in Spain (CI:-0.0503, 95% CI -0.1083, -0.0018) and Sicilia in Italy (CI:-0.0466, 95% CI -0.0999, -0.0148) (Supplementary file 6).

**Table 1:** Inequality measures (Gini coefficient and concentration Index) of 13 European countries across waves 4 to 8.

Gini coefficient				
Country	Wave		Gini coefficient%	95% Confidence Interval (95%CI)
EU 13	4		21.9	(19.0, 24.4)
EU 13	5		19.2	(17.1, 21.2)
EU 13	6		20.8	(18.1, 23.1)
EU 13	7		21.3	(18.6, 23.8)
EU 13	8		22.5	(19.4, 25.2)

Country	Wave		Education Concentration Index	95% Confidence Interval (95%CI)
EU 13	4		-69	(-81, -55)
EU 13	5		-69	(-80, -58)
EU 13	6		-65	(-77, -55)
EU 13	7		-74	(-89, -63)
EU 13	8		-59	(-81, -40)

Country	Wave		Income Concentration Index	95% Confidence Interval (95%CI)
EU 13	4		-44	(-59, -30)
EU 13	5		-66	(-78, -54)
EU 13	6		-74	(-88, -61)
EU 13	7		-79	(-92, -66)
EU 13	8		-62	(-80, -46)

Country	Wave		Urbanization Concentration Index	95% Confidence Interval (95%CI)
EU 13	4		10	(-2, 21)
EU 13	5		14	(5, 22)
EU 13	6		16	(6, 24)
EU 13	7		15	(5, 25)
EU 13	8		8	(-6, 22)

The CI of income for EU 13 was similar to educational level as it was negative and statistically significant. Some of the highest CI were found in Czechia for wave 8 (CI: −0.0278; 95% CI −0.0411, − 0.0166), indicating DM is concentrated in low incomes. Few CIs stood out as the lowest and are found in Switzerland wave 5 (CI:-0.0018, 95%CI -0.0032, -0.0005) and Estonia in wave 5 and 6 (CI:-0.0021, 95% CI -0.0040, -0.0004) and (CI:-0.0023, 95% CI -0.0040, -0.0007), respectively, Fig. 4. Some NUTS regions presented positive values indicating that DM is concentrated in high income individuals as pomurska statistična regija in Slovenia (CI:0.0191, 95% CI 0.0007, 0.0593) in wave 5 and 4 (CI:0.0162, 95% CI 0.0023, 0.0419) and the region Sardegna in Italy (CI:0.0128, 95% CI 0.0018, 0.0358). The highest negative concentration-high concentration in low income- was present in Střední Čechy (CI: -0.1051, 95% CI -0.2283, -0.0063).

Most countries presented non-significant CI for urbanization (Fig. 4). Significant CI in terms of urbanization was small but positive, indicating more concentration of DM in urban area than rural areas. The smallest CI for urbanization was present in Estonia (CI:0.0012, 95% CI 0.0003, 0.0043). Regarding NUTS level, the highest positive concentration is present in Slovenia (CI:0.0310, 95% CI 0.0008, 0.0978) indicating high concentration in urban areas. Negative CI was also present, and the highest region is primorsko-notranjska statistična regija (CI: -0.0185, 95% CI -0.0646, -0.0011), followed by Provincia Autonoma di Trento (CI: -0.0165, 95% CI -0.0533, -0.0015) indicating concentration of DM in rural areas.

#### Multilevel logistic regression model

Descriptive statistics of variables, including frequency, relative frequency and the mean of income are available in Supplementary file 7. Individuals with primary or less level of education have 130% higher odds of having DM, while individuals with secondary education have 45% higher odds of DM compared with those with tertiary education (OR: 2.303; 95% CI 2.301, 2.305; *p* < 0.001 and OR: 1.496 ; 95% CI 1.495, 1.498; *p* < 0.001, respectively). Regarding age groups, a strong gradient was observed. Odds of having DM increased greatly with increase age group, with the highest odds in 80–89 (OR: 2.599; 95% CI 2.597, 2.602; *p* < 0.001) and then decline for the 90 + age group. Living in urban areas was associated with 16% higher odds of having DM compared with living in rural areas (OR: 1.169; 95% CI 1.168, 1.170, *p* < 0.001), Table 2.

**Table 2:** Multilevel Logistic Regression of individuals having diabetes mellitus and country as random effect.

Variable	Odds ratio	95% Confidence Interval	*p*-value
Sex: (Ref: Female)			
Male	1.451	(1.450, 1.451)	<0.001
Education attainment (Ref: Tertiary)			
None/Primary	2.303	(2.301, 2.305)	< 0.001
Secondary	1.496	(1.495, 1.498)	< 0.001
Age group (Ref: 50–59)			
60–69	1.740	(1.739, 1.742)	< 0.001
70–79	2.506	(2.504, 2.507)	< 0.001
80–89	2.599	(2.597, 2.602)	< 0.001
90 and above	1.829	(1.825, 1.832)	< 0.001
Urbanization: (Ref: Rural)			
Urban	1.169	(1.168, 1.170)	< 0.001
Income	0.99998971	(0.99998996, 0.9999898)	
Wave (Ref: Wave 4)			
Wave 5	0.992	(0.991, 0.993)	< 0.001
Wave 6	1.023	(1.022, 1.024)	< 0.001
Wave 7	1.036	(1.035, 1.036)	< 0.001
Wave 8	1.020	(1.019, 1.020)	< 0.001
Level	Variance	SD	
Country (intercept)	0.0532	0.231	
ICC	0.017		

ICC- intraclass correlation coefficient, SD- standard deviation.

## Discussion

This study provides one of the first multi-wave (2010–2020) assessments of DM prevalence inequality at subnational NUTS level across a fixed set of 13 European countries using harmonized SHARE measures for population over 50. We jointly quantify (i) within-country geographic inequality in DM prevalence (GC across NUTS regions) and (ii) socioeconomic-related inequality (CI by income, education, and urbanization), and (iii) assess robustness to underdiagnosis using an IDF-based correction scenario and finally (iv) assess the odds of DM through multilevel model including selective risk factors. Overall, our results align with prior European evidence showing a persistent socioeconomic gradient in DM, with higher burden among lower-income and lower-educated groups, while extending this literature by quantifying within-country heterogeneity at NUTS level across multiple waves. Although the magnitude of geographic inequality was generally modest, it was not uniformly stable: several countries showed gradual increases in regional dispersion over time, whereas others remained largely unchanged. This indicates that stable national prevalence can coexist with widening subnational gaps, which may be missed by country-average monitoring alone. Together, these findings support more geographically targeted prevention and screening strategies focused on socioeconomically disadvantaged populations.

Our prevalence analysis, including 13 European countries, demonstrated that the DM prevalence decreased from 2010 to 2019 (11–10.8%). However, some of the countries showed an alarming increase in the prevalence over the period: Belgium, Czechia, Denmark, Estonia, Slovenia, and Sweden. The increasing prevalence of DM is not a recent phenomenon, as multiple studies have consistently reported data illustrating a rise in the occurrence of this metabolic disorder in Europe^7,9,29^. Comparing our results with Global Burden of Disease (GBD) database for the same countries for age group 55+^30^, the results were consistent in temporal changes in majority of countries, however, our results showed lower estimates than reported in GBD. This could be due to different sources of databases used in formulating GBD results^30^, while our estimates used self-reported information that tend to underestimate prevalence. According to the IDF Diabetes Atlas 2025, the prevalence of DM among individuals aged 20–79 years is 13%^28^. The report also indicates that 33.6% of DM cases in the European region remain undiagnosed. Considering this substantial proportion of undiagnosed cases, our adjusted prevalence estimate may more closely align with findings from the GBD study and the IDF 2025 report. However, this still does not compensate for all cases such as hospitalized cases. A systematic review including 10 EU member states in their prevalence analysis showed that the prevalence increased in all the studies reporting longitudinal data between 1978 until 2017, ranging from 2.82% in 2010 in Denmark to 12.1% in 2017 in France^29^.

In our study, Czechia and Spain had the highest DM prevalence rates during the whole period, from 13.3% (Spain in wave 4) the highest in the base wave to the highest prevalence in the sample: 16.7% (Czechia in wave 8). A few studies based on surveys between 2006 and 2018 reported that DM prevalence in Czechia was high due to factors such as level of education, living in towns/suburbs/rural areas, BMI, waist circumference and hypertension^31–33^. A cross-sectional study performed in 2009/10 reported a prevalence of 13.8% in Spain, and the multivariate logistic regression showed an association of this high prevalence to factors such as higher age, low educational level, obesity and family history of DM^34^.

The lowest DM prevalences were observed in Denmark and Switzerland; 6.0% in Switzerland and 6.7% in Denmark in wave 4 and 6.7% in Switzerland and 7.7% in Denmark in wave 8. These values stood out by far in relation to other countries, not coincidentally, the two countries represent the highest GDPs per capita among the countries included in the study, which can explain less exposure to certain individual and environmental risk factors. A study covering a period between 1996 and 2016 in Denmark reported a slow increase in prevalence over the years, and an important decrease in mortality of DM individuals^35^; this can also explain the low prevalence found in our study. A repeated cross-sectional study investigated the prevalence of DM in an urban region of Switzerland between 2005 and 2017. They aimed to analyze trends in both total and diagnosed DM rates. The findings reveal a consistent prevalence of DM over the specified time frame, decreasing from 9.6% to 8.6%^36^. In our study, a similar, but lower, prevalence between 5.9% and 6.6% within 2010 and 2019 was found; however, the trend in the present study is increasing across time. The same authors published another study reporting inequalities in prevalence of DM between 2005 and 2017 in Geneva (Switzerland) according to income and education. The inequalities in socioeconomic inequalities in total DM prevalence increased between 2005 and 2017^37^.

Geographic inequality in DM prevalence was generally modest in magnitude across countries, but trends were heterogeneous, with some settings showing increases over time, it ranged between 5.8% (the Netherlands in wave 5) to 30.1% (Czechia in wave 8). Furthermore, the DM prevalence is reported to be heavily concentrated in individuals with lower income and low educational levels. Interestingly, the DM prevalence seems to be more concentrated in individuals living in urban areas, but the values were close to zero and non-statistically significant. The increased risk of developing DM in urban areas may be attributed to factors such as sedentary lifestyles, unhealthy dietary patterns, and the impact of urbanization on metabolic health^38^. Health inequalities in DM are pervasive across regions. The differences in the prevalence of DM among subnational European areas result from complex interplays between socioeconomic, cultural, genetic, and healthcare factors. This should encourage the setting up of targeted prevention activities for those at high risk, such as those with low educational attainment and low incomes.

In our present study, the CIs indicated that Spain and Czechia exhibited the highest DM prevalence rates among individuals with the lowest income levels, while Spain also showed the highest DM prevalence observed among individuals with lower educational levels. However, a thorough investigation into the population’s socioeconomic and demographic characteristics, healthcare utilization patterns, and regional or geographic differences in health outcomes is required to fully understand the observed inequality patterns in these countries. In Spain, existing evidence suggests that socioeconomic inequalities influence access to preventive care and DM management services, potentially contributing to the higher burden observed among disadvantaged groups^39^. Similarly, in Czech Republic, differences in income distribution and access to healthcare resources across regions may partly explain the pronounced inequalities observed in DM prevalence^40^.

Our research found that Hamburg and Berlin, Germany’s largest cities, exhibited a high CI for income, suggesting a greater prevalence of DM among wealthy individuals. The literature assessing the association between socioeconomic factors and health status is diverse, particularly in the analysis of subnational regions in Germany. A study including Berlin, Hamburg, and Munich found that homelessness and housing exclusion were not associated with a higher likelihood of chronic diseases and multi-morbidity onset^41^. Interestingly, another study revealed higher rates of DM risk factors such as hypertension, active smoking, and obesity in Northern Germany when compared to the Southern region. Additionally, in 2011, Bavaria, Hamburg, and Berlin had the highest numbers of registered general practitioners per 100,000 residents^42^. In Denmark, regions such as Hovedstaden and Nordjylland reported a higher prevalence among individuals with lower educational levels. The opposite was found in Luxembourg (Belgium), which displayed a contrasting trend, with a higher prevalence of DM concentrated among individuals with higher educational attainment. Individuals of higher socioeconomic levels in Northern region of Denmark seem to have increased healthcare utilization when compared to other regions^43^. Also, people with a higher educational level have more extensive use of out-patient services, rehabilitation and specialists in primary care. However, our regression model indicated increase of odds of DM with low education levels compared with Tertiary education and increase of odds of the disease while living in urban areas. The Principality of Asturias in Spain demonstrated a higher prevalence of DM in urban settings. A study in Spain revealed a pro-poor inequality, favoring economically advantaged individuals in accessing and using General Practitioner services. Even when healthcare needs are similar, elderly individuals with lower incomes do not receive equitable treatment^44^.

Healthcare systems in Western countries are often regionally organized, leading some areas to offer better or more accessible healthcare services to different socioeconomic level populations. In order to understand and address health inequalities between regions, the concepts of ‘pro-poor’ and ‘pro-rich’ health system strategies are essential^45^. To create more equitable and just health systems, policymakers can examine the distribution of health-related resources and the impact of policies on different socio-economic groups. Encouraging regional governance can be an effective response to facilitate regional interventions. Regional governance may allow for more flexibility and responsiveness to local needs and preferences, as well as greater community participation and accountability^46^; may foster innovation and learning across different regions^47^; and it may reduce the burden and complexity of central administration and regulation^48^. The EU, which brings together 27 European countries, provides structural support. The Structural Funds generally support programs in NUTS 2 and NUTS 3 regions, which may provide resources for health-related projects, but health is not in the focus.

The present paper had many strengths mostly related to the fact that we used a large-scale dataset with harmonized data across countries, enhancing the reliability and validity of the study’s findings and ensuring consistent data collection and comparability across diverse populations. Reporting changes in DM across 5 different waves provides a comprehensive view of the disease’s trends over time, offering valuable insights into its progression and potential influencing factors. The inclusion of data on the subnational level is crucial for understanding geographical inequalities within countries. It allows researchers to identify disparities in DM prevalence, healthcare access, and outcomes across different regions, facilitating targeted interventions and policies. The analysis of distribution in inequality data concerning key variables such as educational level, urbanization, and income enriches the study’s depth and comprehension of the social determinants influencing DM prevalence and its unequal impact on different population groups. Performing meaningful inequality analysis using metrics like the GC and CI adds rigor to the study’s examination of DM inequalities. By enabling cross-country comparisons and longitudinal analyses, the study supports researchers and policymakers in identifying best practices, understanding variations in DM management, and assessing the effectiveness of interventions over time.

Some limitations should be reported as a part of the study, even if they are related to SHARE database, study design, statistical analysis, or methodology. First, DM and socioeconomic variables (income, education, and urban/rural residence) are self-reported in SHARE, which may introduce misclassification and recall bias. In particular, self-reported DM captures only diagnosed cases and may underestimate true prevalence due to undiagnosed DM, which has been estimated to represent a substantial share of total DM cases in Europe^28^. In addition, SHARE does not permit confirmation of DM type; although the age profile and treatment patterns suggest that most cases are likely type 2, we cannot reliably distinguish type 1 and type 2 DM. Moreover, severely ill individuals may be underrepresented in survey interviews, which could further underestimate DM prevalence. Second, our analyses are restricted to adults aged ≥ 50 years. Prevalence estimates are presented as crude values within this population and were not age-standardised. Although we adjusted for age in multilevel regression analyses, cross-country and subnational comparisons of crude prevalence may still be influenced by differences in age distributions across countries, regions, and waves. Full age-standardisation using narrow age bands was not feasible at the NUTS level due to sparse age-by-region cells (particularly in smaller NUTS regions and at NUTS 3), which would have resulted in unstable estimates and substantial loss of regional coverage. To aid interpretation, we provide age-stratified prevalence estimates by country and wave in Supplementary file 6. Third, subnational analyses are constrained by small sample sizes in certain NUTS region–wave strata and by missing sampling weights for the Netherlands in waves 6 and 7. Sparse regional strata can yield unstable prevalence estimates with large uncertainty, and these results should be interpreted cautiously. In addition, including different levels of NUTS (1, 2, and 3) in the same analysis may affect the comparability of inequality estimates. Fourth, the study is descriptive and observational, relying on repeated cross-sectional survey waves. As such, the findings reflect associations and do not support causal inference regarding the determinants of DM inequalities. Finally, our inequality analyses focus on three socioeconomic dimensions (income, education, and urban/rural residence). Other relevant factors (e.g., BMI and health behaviours) were not included and may confound observed associations. Results involving urban/rural residence should also be interpreted cautiously because it is a binary, non-ordinal characteristic, and inequality indices based on ranking are less straightforward for such variables.

## Conclusions

In conclusion, the findings of this study demonstrate a slight decrease in the prevalence of DM across European countries from 2010 to 2019, with Czechia and Spain exhibiting the highest prevalence and Switzerland and Denmark registering the lowest. Despite fluctuations in the DM inequality levels over the studied period, the baseline and endpoint levels of inequality remained relatively similar. The most pronounced prevalence of DM was identified among individuals with lower incomes and educational attainment. Across subnational regions, there were diverse levels of inequalities in DM prevalence and associated socioeconomic risk factors. The variability observed in DM prevalence being higher in high-income individuals in some regions and lower in low-income in others suggests a regional inclination toward either pro-poor or pro-rich healthcare. This trend is mirrored in the contexts of urbanization and educational attainment within these regions. It is necessary that policymakers prioritize the implementation of comprehensive strategies aimed at stopping the increasing prevalence of DM. Initiatives focused on prevention and improved healthcare access should be specifically tailored to address the needs of individuals with low socioeconomic levels and limited educational backgrounds. By targeting these vulnerable groups, there is an opportunity to mitigate health inequalities and foster a more equitable distribution of resources and support. The observed variations in prevalence across subnational regions emphasize the need for targeted interventions that consider regional nuances and specificities. This highlights the urgency of addressing social determinants of health to curtail the disproportionate impact of DM on vulnerable populations.

## Supplementary Information

Below is the link to the electronic supplementary material.

Supplementary Material 1

## Data Availability

Data are available in a public, open access repository (share-eric.eu/data/). The data that support the findings of this study are available from the corresponding author upon reasonable request.

## Abbreviations

DM: Diabetes mellitus
EU: European Union
NUTS: Nomenclature of territorial units for statistics
SHARE: Survey of health, ageing and retirement in Europe
GC: Gini coefficient
CI: Concentration index
BMI: Body mass index
GDPs: Gross domestic product
